# Development of predictive nomograms for clinical use to quantify the risk of isolating resistance prone organisms in patients with infected foot ulcers

**DOI:** 10.1017/S0950268818003667

**Published:** 2019-03-20

**Authors:** A. Farkas, F. Lin, K. Bui, F. Liu, G. L. An, A. Pakholskiy, C. F. Stavropoulos, J. C. Lantis, A. Yassin

**Affiliations:** 1 Department of Pharmacy, Mount Sinai West Hospital, New York, NY, 10019, USA; 2 Department of Pharmacy, Mount Sinai St. Luke's Hospital, New York, NY, 10025, USA; 3 Division of Infectious Diseases, Department of Medicine, Mount Sinai West and St. Luke's Hospitals, New York, NY, 10019, USA; 4 Division of Vascular and Endovascular Surgery, Mt. Sinai West and St. Luke's Hospitals, New York, NY, 10019, USA

**Keywords:** Diabetes, foot ulcer, MRSA, *Pseudomonas aeruginosa*, resistance, stewardship

## Abstract

*Pseudomonas aeruginosa* and methicillin-resistant *Staphylococcus aureus* (MRSA) have been considered prevalent pathogens in foot infections. However, whether empiric therapy directed against these organisms is necessary, and in whom to consider treatment, is rather unclear. The aim of this study was to develop predictive algorithms for forecasting the probability of isolating these organisms in the infected wounds of patients in a population where the prevalence of resistant pathogens is low. This was a retrospective study of regression model-based risk factor analysis that included 140 patients who presented with infected, culture positive foot ulcers to two urban hospitals. A total of 307 bacteria were identified, most frequently MRSA (11.1%). *P. aeruginosa* prevalence was 6.5%. In the multivariable analysis, amputation (odds ratio (OR) 5.75, 95% confidence interval (CI) 1.48–27.63), renal disease (OR 5.46, 95% CI 1.43–25.16) and gangrene (OR 2.78, 95% CI 0.82–9.59) were identified as risk factors associated with higher while diabetes (OR 0.07, 95% CI 0.01–0.34) and Infectious Diseases Society of America infection severity >3 (OR 0.18, 95% CI 0.03–0.65) were associated with lower odds of *P. aeruginosa* isolation (*C* statistic 0.81). Similar analysis for MRSA showed that amputation was associated with significantly lower (OR 0.29, 95% CI 0.09–0.79) risk, while history of MRSA infection (OR 5.63, 95% CI 1.56–20.63) and osteomyelitis (OR 2.523, 95% CI 1.00–6.79) was associated with higher odds of isolation (*C* statistic 0.69). We developed two predictive nomograms with reasonable to strong ability to discriminate between patients who were likely of being infected with *P. aeruginosa* or MRSA and those who were not. These analyses confirm the association of some, but also question the significance of other frequently described risk factors in predicting the isolation of these organisms.

## Introduction

Foot ulcers can be a common and serious problem in patients with diabetes and peripheral vascular diseases (PVDs). According to the International Diabetes Federation, the prevalence of diabetic foot ulcers was 13% in North America in 2017, with a global average of 6.4% [1]. These ulcers are frequently complicated by infection that can lead to hospitalisation, and sometimes, amputations [2].

Bacteriology of these ulcers, especially those associated with diabetes, are often polymicrobial, with methicillin-resistant *Staphylococcus aureus* (MRSA) and coagulase negative staphylococci being the most frequently isolated. An increase in the occurrence of multi-drug-resistant organisms (MDRO), like MRSA and *Pseudomonas aeruginosa*, in this population has also been reported [3–7]. MRSA has been isolated from about 23–50% of foot ulcers [3, 6, 8, 9]. *P. aeruginosa* has been considered a common pathogen in foot infections, with prevalence of 4.5%–31% in patients with diabetic ulcers [3, 6, 8, 10]. While *P. aeruginosa* is reported in many patients, it is often a nonpathogenic coloniser when isolated from wounds.

Although foot infections are commonly encountered by clinicians, there is limited guidance on how these patients should be treated, especially when it comes to choosing an empiric antibiotic regimen. The 2012 Infectious Diseases Society of America (IDSA) guideline recommends that empiric therapy against *P. aeruginosa* is usually unnecessary, except when there is a high local prevalence of infections with this organism, in warm climate, and for patients with frequent exposure of the foot to water, conditions that are seldom encountered in the clinical setting at our institutions [9]. According to the IDSA guidelines, empiric therapy against MRSA should be added to patients with a prior history of these infections, when the local prevalence of colonisation or infection is high, or if the infection is clinically severe [9]. Evidence for these recommendations is mostly based on data derived from descriptive studies of the bacteriology of foot ulcers, where the clinicians opportunity to apply the results of these studies to MDRO risk stratification is generally poor, leaving variation in antibiotic therapy for these patients that is mainly based on subjective clinical decision [11]. Past studies have shown that *P. aeruginosa* and MRSA are common in patients with previous hospitalisation, chronic kidney disease, smoking history, prolonged courses of antibiotic therapy, also with amputation, frequent hospitalisation and in those with osteomyelitis [6, 8, 10, 12–15].

Due to the paucity of data for practical application in our area where the rates of bacterial resistance is low, the aim of this study was to evaluate risk factors associated with *P. aeruginosa* and MRSA infections in patients with infected foot ulcers, and to develop predictive algorithms for guidance in estimating the expected probability of these MDRO infections and aid in the empiric selection of an optimal antimicrobial regimen.

## Methods

### Study population

The institutional review board of Mount Sinai Health System waived the requirement for informed consent and granted approval for this study. Adult patients hospitalised at Mount Sinai West and Mount Sinai St Luke's hospitals from June 2015 to June 2016 who presented with infected and culture (from bone, deep tissue fragments and occasional deep tissue swab) positive foot ulcers were included. Clinical diagnosis of infection was based on the presence of at least two of the following criteria: local swelling or induration, erythaema around the ulcer, local tenderness or pain, increase of temperature and purulent discharge. The diagnosis of osteomyelitis was based on imaging following guideline recommendations [9]. Aside from the baseline demographic and laboratory parameters [6, 7, 8, 13], the following risk factors previously evaluated for association with *P. aeruginosa* and MRSA in foot ulcers were identified from the existing literature and were collected retrospectively from the medical record: HbA1c [6, 13, 14], presence of gangrene [8, 13], prior hospitalisation or nursing home stay [5, 12, 16], history of antibiotic usage prior to hospitalisation [5, 6, 8, 13, 14], antibiotic use during the admission prior to culture collection, ulcer size (expressed in cm^2^) and IDSA infection severity classification [8, 13], history of amputation [8], concomitant disease (which refer to the history or clinical records of the patient including coronary heart disease, cerebrovascular disease, renal diseases, immunosuppression, diabetes, and vascular insufficiency [6, 7, 8, 13, 15]), osteomyelitis [8, 10, 12–14], tobacco use [6, 7, 14] and history of prior colonisation or infection with an MDRO [3, 5, 16]. Additionally, baseline erythrocyte sedimentation rate and C-reactive protein, baseline serum lactate level, presence of sepsis on admission, ICU length of stay (LOS) and the Charlson comorbidity index (risk factors that are seldom or not at all reported in the past but probable in the population of patients with infected foot ulcers), were also included in the data collection.

If no antibiotic was given, or the antibiotic administered prior to culture collection was lacking known spectrum of activity against the microorganism eventually isolated, the microorganism was considered not to have been covered by antibiotics empirically. ‘Susceptibility match’ was defined as empirical antibiotic treatment given before obtaining the specimen that subsequently was found to match the susceptibilities of the pathogens recovered from the culture.

### Microbiological studies

Clean culture specimens were collected either at the time of the ulcer debridement or as part of the procedure during amputation of the infected area, where samples from all amputations below the knee were considered. Samples were taken from the infected area following the debridement and povidone iodine cleansing of the tissue that surrounded the wound. Fragments obtained by deep tissue biopsy and bone debridement during surgery served as the specimen for most cases, there were also some specimens that were obtained as deep tissue swabs following guideline recommendations for collection of high quality swab cultures [9]. Bacteria were identified and susceptibility testing was done using the VITEK^®^ 2 automated system (bioMérieux, France).

### Statistical analysis

Descriptive statistics include frequency, median and inter-quartile range where between group comparisons were established using the *χ*^2^ test and the Kruskal–Wallis test, as appropriate. Plausible predictors of MDRO were identified after reviewing the available literature (for details of specific variables considered see under section ‘Study population’). Thereafter, we used the predictors and created univariable and multivariable logistic regression models to identify baseline subject and treatment characteristics that were independently associated with the before mentioned outcomes of interest (identification of *P. aeruginosa* or MRSA in the cultures of infected foot ulcers). We used the R^®^ package glmulti, which does automated model selection, to generate models of all possible combinations and selected the final model based on Akaike's information criterion (AIC) scores and the rules of parsimony [17]. The advantages of this information criteria based approach over the classic stepwise approach to model selection is twofold: first, it allows for the identification of the ‘best’ model (based on the AIC in our case) through evaluation and comparison of all the possible models that can be constructed from a set of candidate predictor variables; and second, it allows for the assessment of model-selection uncertainty and multi-model inference. Final model evaluation for *P. aeruginosa* was based on the Hosmer–Lemeshow statistic, while for the MRSA model, the Cessie–van-Howelingen–Copas–Hosmer unweighted sum of squares statistic was used due to the low number of groups identified by the Hosmer–Lemeshow goodness of fit test [18, 19]. In the final models selected, all effects were considered significant with a *P* value of <0.05. Next, we further evaluated the final model's performance using bootstrapping. Once bootstrapping procedure was repeated 1000 times, an average *C* statistic for the bootstrap sample was calculated. This average *C* statistic was then subtracted from the *C* statistic developed from the original sample to calculate model optimism. A concern was that as models are developed to provide the best fit for the data, there is the possibility that a model will be over fitted, resulting in an optimistic assessment of the model's predictive ability as compared to predictions that would be based on an external dataset. To adjust for this effect, the observed performance was calculated by subtracting the optimism from the apparent performance of the models. To select the optimal cut-off point in defining a positive test, the probability cut-off that gives the maximum Youden's index was chosen [20]. Model sensitivity, specificity, positive (PPV) and negative (NPV) predictive values were also calculated. All statistical analyses were performed with the R^®^ software and applicable packages [21, 22].

## Results

Demographic and clinical features of the 140 patients in the study are summarised in Table 1. Overall, 67.9% of the patients were male with a median (interquartile range – IQR) age of 64 (56, 73) years. Of the patients, 37.9% had IDSA infection severity >3, 30.2% had PVD, and 26.4% had evidence of gangrene on presentation. When compared to the patients without diabetes (*n* = 31 (22.2%)), patients with diabetes (*n* = 109 (77.8%)) had higher Charlson scores (3 *vs.* 5, *P* = 0.002), were more likely to have PVD (9.6% *vs.* 36.1%, *P* = 0.009), had higher median HbA1c levels (5.8% *vs.* 10.0%, *P* < 0.001) and were more likely to have an amputation (12.9% *vs.* 46.3%, *P* = 0.002). In the population studied, 119 (85%) of the patients received at least one dose of antibiotic therapy prior to culture collection with a median time between the first dose of antibiotic and culture collection of less than 1 day (IQR 0, 1) days, and 113 (94%) of them also met the definition of covered with the empirically administered antibiotic for at least one of their isolated organism.

**Table 1.** Baseline demographics and comorbid conditions in the chronic foot infection population surveyed

Variable (%)	Total	No DM	Yes DM	*P* value
*N*, (%)	140	31 (22.2)	109 (77.8)	–
Gender				
Male	67.9	61.3	69.7	0.503
Age (years)	64 (56, 73)	70 (61, 76)	62 (55, 72)	0.028
IDSA infection severity >3	37.9	32.3	39.4	0.604
Charlson comorbidity index score	4 (3, 6)	3 (2.5, 4)	5 (3, 6)	0.002
Gangrene	26.4	16.1	29.4	0.214
Previous admission	17.1	25.8	14.7	0.238
*P. aeruginosa*	13.6	22.6	11.0	0.134
MRSA	23.7	23.3	23.9	1.000
PVD	30.2	9.68	36.1	0.009
HbA1c (mmol/mol)	9 (6.8, 10.9)	5.85 (5.65, 6.45)	10 (7.4, 11.1)	<0.001
Immunosuppression	2.86	6.45	1.83	0.213
Amputation	38.8	12.9	46.3	0.002
Antibiotic prior to culture collection	119 (85)	24 (80)	95 (87)	0.294
Total days of inpatient antibiotics	8 (6, 12)	7 (5.5, 8)	9 (6, 13)	0.011
LOS in hospital (days)	9 (6, 13)	8 (6, 10.5)	9 (7, 15)	0.074

DM, diabetes mellitus; MRSA, Methicillin-resistant *Staphylococcus aureus*; PVD, peripheral vascular disease; HbA1c, haemoglobin A1c.

*N* is the number of patients. Data are shown as the median (interquartile range) or percentage of total. Immunosuppression is defined as HIV+, active chemotherapy or chronic high dose steroids.

Results of the microbiological assessment are given in Table 2. A total of 307 bacteria were isolated, where the majority of cultures (67.9%) were found to be polymicrobial. Overall, 212 (69.1%) of the isolates met the definition of susceptibility match. The most frequently isolated bacteria were MRSA (11.1%), followed by *Streptococcus agalactiae* (8.7%), and *Enterococcus* species (8.4%). *P. aeruginosa* was ranked the fifth most common organism identified (6.5%).

**Table 2.** Specimen description and overall prevalence of pathogens from the studied population

Variable	Total, *n* (%)
Type of specimen included from subjects	
Bone	55 (39.3)
Tissue biopsy	70 (50)
Swab^a^	15 (10.7)
Bacteria	
Gram-positive bacteria	
* *Methicillin-sensitive *Staphylococcus aureus*	24 (8.0)
* *Methicillin-resistant *Staphylococcus aureus*	34 (11.1)
* S. agalactiae*	27 (8.7)
* Enterococcus* spp.	26 (8.4)
* *Coagulase negative *Staphylococcus*	10 (3.2)
Gram-negative bacteria	
Non-ESBL producing *Escherichia coli*	10 (3.2)
ESBL producing *E. coli*	7 (2.2)
* P. aeruginosa*	20 (6.5)
* Proteus mirabilis*	19 (6.1)
* Prevotella bivia*	16 (5.2)
* Morganella morganii*	8 (2.6)
Anaerobic bacteria	
*Bacteroides* spp.	13 (4.2)
Other, Gram-positive bacteria	
Aerobe	25 (8.2)
Anaerobe	20 (6.6)
Other, Gram-negative bacteria	
Aerobe	37 (12.2)
Anaerobe	11 (3.6)
Total	307

ESBL, extended spectrum beta-lactamase.

a Swab specimens were collected according to recommendations in ref. [9].

In the univariable analysis, IDSA infection severity >3, presence of gangrene and amputation were associated with *P. aeruginosa*, *vs.* history of MRSA infection and amputation were associated with MRSA (Table 3). Outcomes of the multivariable logistic regression analysis showed that in the final model (Table 4) a history of renal disease (odds ratio (OR) 5.46, 95% confidence interval (CI) 1.43–25.16) and a history of amputation (OR 5.75, 95% CI 1.48–27.63) at the time of culture were significant predictors of infection with likely isolation of *P. aeruginosa.* Having diabetes (OR 0.07, 95% CI 0.01–0.34) or a wound classified as IDSA infection severity of >3 (OR 0.18, 95% CI 0.03–0.65) contributed to lower odds of sequestering the organism. The presence of gangrene showed borderline significance (OR 2.78, 95% CI 0.82–9.59) in influencing the odds of pseudomonal infections. The final model showed reasonable discrimination (*C* statistic (area under the receiver operating characteristic curve) 0.85). Validation via 1000 bootstrap samples resulted in an optimism-corrected *C* statistic of 0.81. Specificity and sensitivity of the model were 74.3% and 89.4%, respectively. PPV and NPV of the model were 35.4% and 97.8%.

**Table 3.** Results of the univariable analysis of potential risk factors

Predictor	*P. aeruginosa*			MRSA		
	OR	95% CI	*P* value	OR	95% CI	*P* value
Gender, male	0.78	0.29–2.25	0.64	0.62	0.26–1.51	0.28
Weight	0.99	0.96–1.01	0.29	0.98	0.96–1.00	0.19
Initial lactate	0.96	0.37–1.71	0.90	1.49	0.90–2.62	0.11
Ulcer surface area (cm^2^)	1.08	0.99–1.21	0.11	0.97	0.83–1.07	0.72
IDSA infection severity >3	0.27	0.06–0.85	**0.04**	0.50	0.18–1.25	0.15
Initial ESR	1.00	0.98–1.02	0.98	1.00	0.99–1.02	0.23
Initial CRP	1.00	0.98–1.01	0.68	1.00	0.99–1.01	0.30
Gangrene	2.99	1.09–8.15	**0.03**	0.57	0.18–1.54	0.30
Previous admission	1.92	0.57–5.70	0.26	1.50	0.49–4.09	0.43
Sepsis on admission	0.59	0.13–1.94	0.43	0.72	0.22–1.96	0.55
PVD	1.08	0.35–2.96	0.89	1.28	0.54–3.11	0.58
Vascular insufficiency	1.52	0.46–4.44	0.46	0.64	0.17–1.87	0.45
DM	0.42	0.15–1.24	0.10	0.99	0.37–2.94	0.99
Renal disease	2.40	0.88–6.48	0.08	1.21	0.47–2.91	0.67
Amputation	3.11	1.16–8.91	**0.03**	0.37	0.12–0.94	**0.04**
ICU LOS	0.95	0.48–1.24	0.80	0.99	0.67–1.24	0.98
Charlson comorbidity index score	1.20	0.99–1.46	0.06	0.95	0.78–1.14	0.61
HbA1c	0.81	0.49–1.17	0.31	1.09	0.83–1.43	0.52
Smoking history	1.43	0.31–5.02	0.60	1.91	0.56–5.75	0.26
Nursing home resident	0.11	0.00–1.57	0.13	1.8	0.47–5.25	0.28
History of *P. aeruginosa* infection	2.73	0.37–13.82	0.25	0.68	0.03–4.25	0.73
History of MRSA infection	0.56	0.03–3.13	0.58	5.09	1.46–17.82	**<0.01**
Prior antibiotic course	2.08	0.67–5.9	0.18	1.18	0.39–3.14	0.74
Osteomyelitis	0.83	0.31–2.20	0.70	1.79	0.76–4.38	0.18
Immunosuppression	0.35	0.01–7.57	0.51	0.27	0.01–5.41	0.40

ESR, erythrocyte sedimentation rate; CRP, C-reactive protein; PVD, peripheral vascular disease; DM, diabetes mellitus; LOS, length of stay; HbA1c, haemoglobin A1c; MRSA, Methicillin-resistant *Staphylococcus aureus*.

Statistically significant *P* values are in bold.

Immunosuppression is defined as HIV+, active chemotherapy or chronic high dose steroids.

**Table 4.** Summary of the top five best fit models for *P. aeruginosa* and MRSA

*P. aeruginosa* models	AIC	LL	LR	LR *P* value
**IDSA infection severity >3 + gangrene + DM + renal disease + amputation**^a^	95.75	−41.87	NA	NA
IDSA infection severity >3 + gangrene + amputation + DM + renal disease + sepsis	96.56	−41.27	1.19	0.27
IDSA infection severity >3 + gangrene + DM + renal disease + amputation + immunosuppression	96.57	−41.28	1.18	0.27
IDSA infection severity >3 + gangrene + DM + renal disease + amputation + history of *P. aeruginosa* infection	96.88	−41.44	0.86	0.35
IDSA infection severity >3 + gangrene + DM + renal disease + amputation + prior antibiotic course	96.74	−41.37	1.00	0.31
**MRSA models**				
**Osteomyelitis + amputation + history of MRSA infection**^b^	130.9	−61.46	NA	NA
Osteomyelitis + amputation + history of MRSA infection + nursing home	132.2	−61.08	0.76	0.38
Osteomyelitis + amputation + history of MRSA infection + smoking history	132.1	−61.05	0.80	0.36
Osteomyelitis + amputation + history of MRSA infection + IDSA infection severity >3	131.8	−60.95	1.10	0.29
Osteomyelitis + amputation + history of MRSA infection + PVD	132.4	−61.11	0.52	0.46

Final models are in bold.

AIC, Akaike's information criteria; LL, log-likelihood; LR, likelihood ratio, all presented *vs*. the final model; DM, diabetes mellitus; PVD, peripheral vascular disease.

Immunosuppression is defined as HIV+, active chemotherapy or chronic high dose steroids.

a Hosmer–Lemeshow *χ*^2^ statistic = 10.05, degrees of freedom = 8, *P* = 0.26.

b Cessie–van-Howelingen–Copas–Hosmer goodness-of-fit test sum of squared errors = 19.6; expected value = 19.4; standard deviation = 0.19; studentised test statistic = 1.32, *P* value = 0.18.

**Fig. 1.**
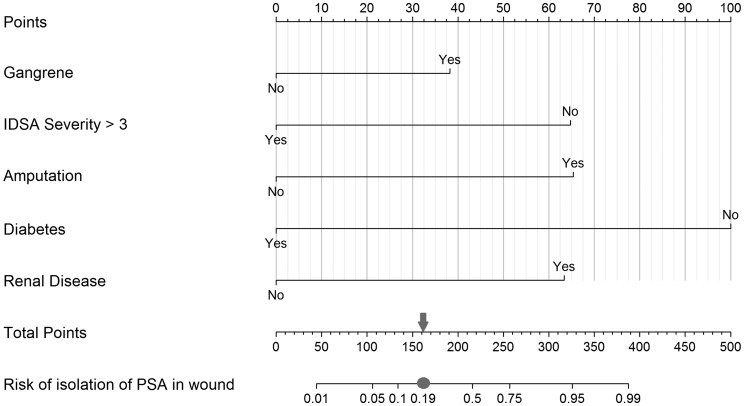
Nomogram to predict risk of isolation of *P. aeruginosa* (PSA) in the infected wound. Each predictor with the presence (‘Yes’) or absence (‘No’) of the condition can be mapped to the Points axis on top of the nomogram to determine how many points towards the predicted probability of PSA in the wound the patient receives for the particular condition. Then, the sum of all of these points can be referred to in the Total points axis. Last, based on the Total points, the probability of isolating PSA in the wound can be obtained by drawing a straight line down to the corresponding Risk of isolation of PSA in wound axis. As an example, a patient presenting with gangrene (38 points for ‘Yes’ or 0 for ‘No’ on the Gangrene axis), an IDSA severity category of 3 (65 points for ‘No’ or 0 for ‘Yes’ on the IDSA Severity >3 axis), with amputation (66 points for ‘Yes’ or 0 for ‘No’ on the Amputation axis), who is not a diabetic (100 points for ‘No’ or 0 for ‘Yes’ on the Diabetes axis) and with chronic kidney disease (64 points for ‘Yes’ or 0 for ‘No’ on the Renal disease axis) would achieve a score of 333 (sum of all points for the individual risk factors), which then is referred to on the Total points axis, indicating a probability of isolating PSA in the wound of approximately 0.95 on the Risk of isolation of PSA in wound axis. In context with the measures of our model's discriminative ability, the case is then categorised into a predicted positive for PSA isolation in the wound when the estimated probability equals to or exceeds 0.19 (grey circle, which equals to 162 on the Total Points axis indicated by the grey arrow) *vs*. a negative for PSA isolation in the wound when the calculated probability is below the value of 0.19.

**Fig. 2.**
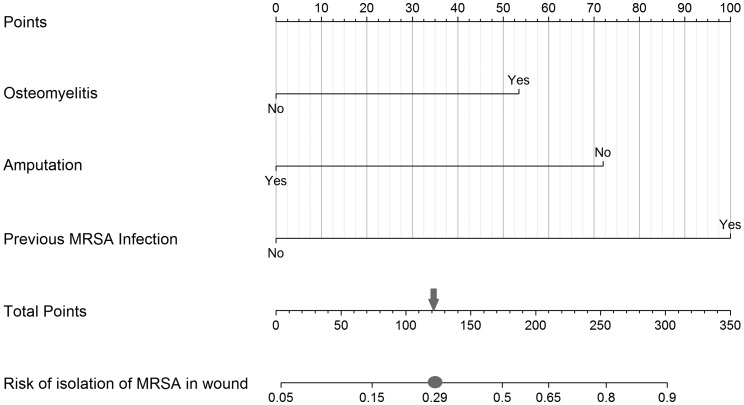
Nomogram to predict risk of isolation of MRSA in the infected wound. To establish risk of isolation of MRSA in the wound, the steps outlined in Figure 1 should be followed using the predictors and respective point values assigned to the presence or the absence of a condition (53 points for ‘Yes’ or 0 for ‘No’ on the Osteomyelitis axis; 72 points for ‘No’ or 0 for ‘Yes’ on the Amputation axis; and 100s point for ‘Yes’ or 0 for ‘No’ on the Previous MRSA infection axis). Then, after summing the individual point values to establish the value for total points, categorise the case into a predicted positive for MRSA isolation in the wound when the estimated probability equals to or exceeds 0.29 (grey circle which equals to 122 on the Total points axis indicated by the grey arrow) *vs.* a negative for MRSA isolation in the wound when the calculated probability is below the value of 0.29.

Similar analysis for MRSA in the final model established osteomyelitis (OR 2.52, 95% CI 1.00–6.79) and history of infection with MRSA (OR 5.63, 95% CI 1.56–20.63) as dominant risk factors in predicting the presence of this bacterium, while amputation (OR 0.29, 95% CI 0.09–0.79) seemed to present with a decreased odds of MRSA infections (Table 4). The final model demonstrated fair discrimination showing a *C* statistic of 0.72, and an optimism-corrected statistic of 0.69. The specificity and sensitivity of this final model were 72.5% and 59.2%, respectively. PPV and NPV of this model were 34.07% and 88.1%. Graphical nomograms – based on the logistic regression models developed – to approximate the risk of isolating these two resistance prone organisms are presented in Figures 1 and 2.

## Discussion

The rates of chronic wound MRSA and *P. aeruginosa* infections vary widely between geographic locations and institutions [5, 10]. Within our population, diabetes was identified in the majority of the patients, at a frequency similar to what has been observed by others [5]. We included only cultures from deep tissue and bone allowing for accurate identification of the organisms likely to be the cause of infection [9, 23]. Overall, the makeup of the microbial populations in our study reflected those which have been previously reported, with the most commonly isolated organisms being gram positives, and a little over half of the infections were polymicrobial [6, 8, 12]. The prevalence of MRSA and *P. aeruginosa* was within the lower range of previously reported (5% to 30% and 4.5% to 31%, respectively) and in line with general expectations according to the patient population's geographic location (a non-tropical urban setting in the developed world) served by our institution [5, 15, 16]. While the findings of our analysis from this population confirm the close association of some (amputation, osteomyelitis, renal disease, gangrene), it also questions the significance of other (diabetes) frequently described risk factors used in the decision making of selecting empiric treatment for an MDRO associated foot infection [5–10, 12–15, 16, 24, 25].

Previous publications suggest numerous demographic and clinical patient characteristics – including male gender, smoking history, HbA1c level, diabetes, previous treatment history and amputation – that seem to be strong predictors of the presence of *P. aeruginosa* [6, 7]. Patients that require amputations are often those with the most severe infections and long standing, deep foot ulcers, characteristics of the wound previously linked to identification of *P. aeruginosa* [13, 26]. This subpopulation of patients is also more likely to previously have received multiple courses of broad-spectrum antibiotics, which undoubtedly increases the risk of the emergence of MDROs. In the study by Ertugrul *et al*., patients with history of amputation were 7.2 times more likely to have an MDRO isolated, representing odds comparable to the estimates by us (OR 5.7) [8].

Renal disease, a well-known comorbid condition regularly linked to the presence of an MDRO, was another risk factor we found to significantly increase the odds of *P. aeruginosa* isolation [21]. In particular, chronic kidney disease has been shown to escalate the risk for isolating drug-resistant pathogens [15, 21]. Patients with chronic kidney disease are more susceptible to some infections by opportunistic pathogens as a consequence of altered innate immunity resulting in impaired polymorphonuclear chemotaxis and phagocytosis [27]. Furthermore, given frequent contact with health care settings, patients with chronic kidney diseases have many opportunities for exposure to exogenous organisms, including drug-resistant bacterium [28].

Contrary to popular belief, diabetes, another common comorbid condition in patients with foot ulcers, was not associated with isolation of *P. aeruginosa* in our population. It is speculated that *P. aeruginosa* is more prevalent in diabetic wounds not only because diabetic patients may simply come into contact with it more frequently, but also because there is some selective pressure in the environment of the diabetic wound (insulin mediated changes stimulating biofilm formation) that favours *P. aeruginosa* colonisation and/or persistence [29]. In a study looking at patients with foot ulcers, with and without diabetes, 62.5% of the diabetics had *P. aeruginosa* infections compared to only 37.5% of the non-diabetics [7]. Unlike our approach, the method of sampling in that study included suboptimal culturing from surface swabs, an inferior approach over deep tissue biopsies, which likely influenced their microbiology results [9, 23]. Our analysis repudiates the common misconception that patients with diabetes (*vs*. without diabetes) are at a higher risk for having foot infections caused by this organism.

IDSA infection severity grade >3 was also found to be associated with significantly lower odds of *P. aeruginosa* isolation. Ji *et al*. assessed wound grades, at categories of low and high scores encompassing the full spectrum of the severity of the diseases, and found no significant association between high scores on the Wagner classifications and an MDRO isolation, a finding analogous to our results showing the lack of an increase in risk of these types of infection where wounds are classified as high severity [13].

The presence of gangrene, a not uncommon presentation of pseudomonal infection, was also included in our final regression to ensure optimal predictive performance of the model. Endotoxins and damaging enzymes produced by *P. aeruginosa* often lead to destruction of the skin and invasion of local blood vessels facilitating thrombosis formation, precipitating tissue ischaemia, necrosis and eventually gangrene. Our decision to retain gangrene as a predictor was influenced not only by its plausible relationship to *P. aeruginosa* infections, but also by the results of the *P* values from the univariable (*P* = 0.03), multivariable (*P* = 0.09) and likelihood ratio analysis (model with (LL = −41.87) *vs.* without gangrene (LL = −43.23) LR = 2.72, *P* = 0.09) which – when evaluated on a continuous scale – would leave less than 10% chance for the respective null hypothesis to hold.

The current IDSA guideline's proposition on when to consider empiric therapy against MRSA cited the work published by Eleftheriadou *et al*., who performed a review of 20 published studies and discussed factors noted to increase the risk for infection with MRSA. Within this review, some, but not all of the studies, included prior long-term or inappropriate use of antibiotics, previous hospitalisation, long duration of the foot wound, the presence of osteomyelitis and nasal carriage of MRSA as possible risk factors, with the strongest predictor being previous history of MRSA infection [24]. Our multivariable analysis confirmed that the diagnosis of osteomyelitis is associated with increasing odds of isolating MRSA, a finding well documented in the literature [10, 12]. Ji *et al*. conducted their study to determine MDRO profile in diabetic foot ulcers. In their work, osteomyelitis was also associated with MDROs including MRSA [13]. Prior to that, Ertugrul *et al*. also reported that osteomyelitis increased the risk of MDRO infection by 2.8-fold [8]. In addition, patients with osteomyelitis are more likely to be exposed to longer courses of broad-spectrum antibiotics. The rate and extent of antibiotic penetration into bone tissues – especially in case of poor penetration – may also foster resistant strain development.

We also demonstrated that previous isolation of a MRSA is an additional risk factor for infection with this organism in foot ulcers, findings that are similar to what was established in a study done by Lavery *et al*., where history of a MRSA infection within the past 12 months in the univariable analysis was shown to be significantly associated with developing a future MRSA infection [16]. The ability of MRSA to colonise and infect the skin is mainly dependent on physical and biochemical mechanisms that corrupt host cutaneous defenses. Colonisation is usually limited to skin surface, while infection is generally characterised by the involvement of subcutaneous or deepest tissues. The bacteria can cause infections if they enter the body through cuts, open wounds, or other breaks in the skin, the type of environment readily furnished by foot ulcers.

Having a history of amputation in our study was associated with lower odds of MRSA positive cultures, which is in contrast to what was found by Ertugrul *et al*., where amputation was associated with an increased likelihood of growing a resistant bacteria, including MRSA [8]. This discrepancy seems to be the result of their difference in approach in evaluating the risk factors for MDROs. The outcome variable MRSA (26% of all the resistant bacteria) and other MDROs (74% of all the resistant bacteria) seem to have been classified with the same indicator suggesting that the increase in risk for an MDRO by amputation could have been driven by organisms other than MRSA. Additionally, differences in culturing practices could also have been associated with the relative rate of the identification of certain bacteria. This lower risk of isolating MRSA in the wound associated with amputation is opposite of what we identified in the *P. aeruginosa* model, where amputation increases the likelihood of isolating the organism, a finding that requires further discussion. Gangrene is defined as dead tissue in the foot resulting from inadequate blood flow supply, and is one of the manifestations of critical limb ischaemia and possibly a deep infection, a presentation that often requires immediate surgical intervention with removal of necrotic or poorly vascularised tissue, including the infected bone [30]. In a previous work, Kono *et al*. conducted a retrospective analysis of 116 patients who underwent foot amputation for nontraumatic reasons, with the aims to identify the incidence of and risk factors for ipsilateral reamputation after an initial forefoot amputation. In their multivariable analysis, gangrene on admission was a significant and independent risk factor associated with reamputation (OR 3.81, 95% CI 1.60–9.12) [31]. In our study, patients with *P. aeruginosa* had higher incidence of gangrene, *vs.* the patients with MRSA (48% and 18%, respectively), and patients who had gangrene had higher rates of amputation *vs.* those who did not (67% and 29%, respectively). Our interpretation of these disparities suggests that the opposite findings in the odds related to amputation (higher odds for *P. aeruginosa* and lower odds for MRSA) in our study was driven mainly by the differences in the rates of gangrene requiring amputation in the group of patients whose culture grew *P. aeruginosa vs.* those whose grew MRSA.

The measures of our models’ ability to discriminate cases with and without these MDROs are not perfect, but are comparable to the reported sensitivity and specificity of other risk prediction tools generally used in practice [32, 33]. A tool that has gained significant popularity over the past decade in assessing community onset pneumonia patients’ risk for drug-resistant organisms (MRSA, *P. aeruginosa*, *Acinetobacter* species and *Klebsiella pneumonia*) is the healthcare associated pneumonia criteria (HCAP) [32]. In a validation study, *C* statistic (0.6), specificity (65%) and sensitivity (79%) of the HCAP criteria in accurately identifying those patients that should receive broad antibiotic coverage against drug-resistant organisms were lower than the calculated measures of our *P. aeruginosa* models discriminative ability [34]. Besides the models favourable predictive ability, our nomograms complement clinical judgment and work flow by providing a user friendly interface which eliminates the need for complicated computer software to make predictions and interpret the results.

There are various considerations to bear in mind when interpreting our research results. This was a retrospective study, and the findings are subject to the nuances associated with this type of study design. A large fraction of our patient population received prior antibiotic known to be active against the commonly identified organisms in infected foot ulcers, which could have affected our culture positivity results. A focused review of the susceptibility profile of organisms isolated in this study we feel repudiates this assumption. Our findings indicate that nearly 70% of all the bacteria that eventually grew were exposed to a prior antibiotic that had activity against the subsequently isolated organism. This conclusion is comparable to that reported by Kim *et al*., in the literature, arguing against a significant impact of our pre-treatment regimens on culture positivity [35]. A consequence of retrospective cohort studies using health records is that not all pertinent risk factors are likely to have been identified and subsequently recorded, or recorded accurately. In this work, study data were limited to inpatient records; thus, the independent variables evaluated may not have entirely echoed the influence of the full spectrum of possible predictors on the selected outcomes. Most notably type and duration of ulcers and the number and appropriateness of previous antibiotic courses received, that we were unable to evaluate for association due to the lack of consistent documentation in our data sources, have been demonstrated in previous studies to be significantly correlated with the presence of an MDRO [13]. Our patient population was mixed and relatively small in size, including patients with and without diabetes, making it difficult to extrapolate or compare our results to earlier studies, which were comprised mainly of patients with diabetic foot ulcers. However, we feel analysis of this mixed population as one, resulted in a unique and more real world depiction of the risk analysis that encompass the diversity of patient types often served by the clinicians specialised in the treatment of infected foot ulcers. This approach of including the mixed population was also essential in allowing us to evaluate diabetes independently for its possible association with the isolation of these difficult to treat organisms. Lastly, while internal validation of the models using bootstrapping is an approach well-accepted in the medical literature, external validation of the models predictive performance using an independent but ‘plausibly related’ populations’ dataset is required prior to implementation of the nomograms in the clinical setting.

## Conclusion

In our double centre-based retrospective study, including patient with infected foot ulcers with or without diabetes, amputation and renal diseases were found to be dominant predictors, whereas infection severity classification and diabetes were not associated with the presence of *P. aeruginosa* infections. In the case of MRSA, osteomyelitis and previous history of a MRSA infection showed significant association with positive culture results for this organism, but amputation was associated with lower odds of identifying the bacteria. Our study results and the predictive nomograms provide guidance to local clinicians on assessing the need for broad spectrum empiric antibiotics in patients with infected foot ulcers. Validation of the predictive algorithms in independent datasets of alike patient populations is warranted to establish generalisability of the results.

## Author contributions

All authors had full access to the data in the study and share final responsibility for the decision to submit for publication.
